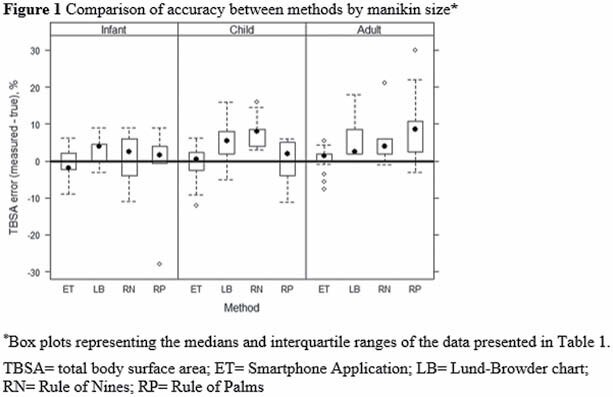# 56 Evaluation of a Smartphone Application as a Method for Calculating Total Body Surface Area Burned

**DOI:** 10.1093/jbcr/irac012.059

**Published:** 2022-03-23

**Authors:** Cindy D Colson, Emily A Alberto, Zachary P Milestone, Nikita Batra, Tyler Salvadore, Hadi Fooladi, Kevin Cleary, Rima Izem, Randall S Burd

**Affiliations:** Children's National Hospital, Bowie, Maryland; Children's National Hospital, Washington, District of Columbia; Children's National Hospital, Washington, District of Columbia; Children's National Hospital, Washington, District of Columbia; Children's National Hospital, Washington, District of Columbia; Children's National Hospital, Washington, District of Columbia; Children's National Hospital, Washington, District of Columbia; Children's National Hospital, Washington, District of Columbia; Children's National Hospital, Washington, District of Columbia

## Abstract

**Introduction:**

Current methods of burn estimation can lead to incorrect estimates of the total body surface area burned, especially among injured children. Inaccurate estimation of burn size can impact initial management, including unnecessary transfer to burn centers and fluid overload during resuscitation. To address these challenges, we developed a smartphone application that calculates the total body surface area of a burn using a body-part by body-part approach. The aims of this study were to assess the accuracy of the smartphone application and compare its performance to three established methods of burn size estimation (Lund-Browder Chart, Rule of Nines, Rule of Palms).

**Methods:**

Twenty-four healthcare providers used each method to estimate burn sizes on moulaged manikins. The manikins represented different ages (infant, child, adult) with different total body surface area burns (small < 20%, medium 20-49%, large >49%). We calculated the accuracy of each method as the difference between the user-estimated and actual total body surface area. We used multivariable modeling to control for manikin size and method.

**Results:**

Among all age groups and burn sizes, the smartphone application had the greatest accuracy for burn size estimation (-0.01%, SD 3.59%) followed by the Rule of Palms (3.92%, SD 10.71%), the Lund-Browder Chart (4.42%, SD 5.52%), and the Rule of Nines (5.05%, SD 6.87%).

**Conclusions:**

The smartphone application may improve the estimation of total body surface area burned compared to existing methods.